# 864. Can Population Risk Factors be Directly Applied to Guide Individual Patient’s Diagnosis and Treatment in Clinical Practice? – the Need to Re-Direct Epidemiology Research for Clinical Application in the Future

**DOI:** 10.1093/ofid/ofad500.909

**Published:** 2023-11-27

**Authors:** Chaorui C Huang, Hong Xu

**Affiliations:** New York City Department of Health and Mental Hygiene, New York; Karolinska Institute, Stockholm, Stockholms Lan, Sweden

## Abstract

**Background:**

The implementation of population risk factor-guided diagnostic approach and treatment regimen has been widely considered in clinical practice to treat individual patient. However, the meaning of these risk factors, which are primarily developed from population-based epidemiology studies and thus presented probability and risk/odds ratio, are hard to interpret in clinical settings for individual patient. In this study, we provided an example of risk analysis for COVID-19 early intervention, in which the diagnostic accuracy of a population risk factor-based treatment regimen and an individual risk factor-based treatment regimen were compared, and the associated benefits and costs were evaluated.

**Methods:**

The treatment regimen guided by the population risk factors was defined as such, that every COVID-19 patient aged ≥65 years old, and/or with at least one of the pre-selected chronic co-morbid conditions will be given a 500 mg/8 mL dose of the monoclonal antibody therapy, Sotrovimab intravenous, to prevent fatal outcome. The individual risk factor-based treatment regimen was determined using logistic regression model. We set the cost of Sotrovimab as $315.00 per patient, and the total cost including medication, hospital and infusion center administration charges, and charges for medical staffs as $2,000.00 per patient.

**Results:**

Our study showed that the treatment regimen guided by population risk factors had resulted in a large amount of patient misclassification, with only 51.9% overall diagnostic accuracy, which is close to a random chance. However, by using the precision approach we developed for individual patient, the diagnostic accuracy was improved to 75%. Population risk factor-based treatment regimen prevented 1,570 more patients from a fatal outcome, however, it added 8 million dollars of cost for Sotrovimab and overall additional 54 million dollars of total cost.

**Conclusion:**

Population level risk analysis provided inadequate information for individual patient’s diagnosis and treatment in clinical practice. Future research needs to focus on developing personalized diagnostic and treatment approach. We anticipate the improved clinical prediction by adding additional laboratory biomarkers to the individual precision diagnostic approach we developed.

**Disclosures:**

**All Authors**: No reported disclosures

